# Male mice are susceptible to brain dysfunction induced by early-life acephate exposure

**DOI:** 10.3389/fnins.2024.1404009

**Published:** 2024-07-10

**Authors:** Takahiro Sasaki, Jahidul Islam, Kenshiro Hara, Tomonori Nochi, Kentaro Tanemura

**Affiliations:** ^1^Laboratory of Animal Reproduction and Development, Graduate School of Agricultural Science, Tohoku University, Aoba-ku, Sendai, Japan; ^2^Laboratory of Functional Morphology, Graduate School of Agricultural Science, Tohoku University, Aoba-ku, Sendai, Japan

**Keywords:** acephate, organophosphate pesticides, central nervous system, neurodevelopmental toxicity, low-dose effects, sex difference, gut microbiota

## Abstract

**Background:**

Acephate is a widely used organophosphate insecticide. Exposure to endocrine-disrupting chemicals, such as acephate, can interfere with neurodevelopment in childhood, increasing the risk of higher brain dysfunction later in life. Furthermore, brain dysfunction may be related to chemical exposure-related disturbances in the gut microbiota. However, the effects of early acephate exposure on the brains of adult males and females as well as on the adult gut environment remain poorly understood.

**Methods:**

This study investigated the effects of perinatal acephate exposure on the central nervous system and gut microbiota of mice, including sex differences and environmentally relevant concentrations. C57BL/6 N pups were exposed to acephate (0, 0.3, 10, and 300 ppm) via the dam in their drinking water from embryonic day (E) 11.5 to postnatal day 14. We examined its effects on the central nervous system of adult males and females.

**Results:**

In the male treatment group, impairments in learning and memory were detected. Immunohistochemical analysis revealed a decrease in SOX2-, NeuN-, DCX-, and GFAP-positive cells in the hippocampal dentate gyrus in males compared to the control group, whereas GFAP-positive cells were fewer in females. In addition, gut microbiota diversity was reduced in both sexes in the experimental group.

**Conclusion:**

Our study demonstrates that the effects of early-life exposure to acephate are more pronounced in males than in females and can lead to a lasting impact on adult behavior, even at low doses, and that the gut microbiota may reflect the brain environment.

## 1 Introduction

During the embryonic, fetal, infant, and adolescent stages, organisms are more susceptible to the effects of chemical exposure than adults. During the maturation process of the central nervous system (CNS), multiple continuous events (i.e., neurogenesis, synaptogenesis, gliogenesis, and synapse pruning) occur at appropriate times (Jiang and Nardelli, 2016). Hence, if these neurodevelopmental mechanisms are disrupted in early life by endocrine-disrupting chemicals (EDCs), the risk of higher brain dysfunction may increase later in life. In particular, neurogenesis in the dentate gyrus (DG) plays a functional role related to anxiety, learning, and memory in the CNS and reduced or increased neurogenesis by various EDCs can lead to brain dysfunction (Martini et al., 2014; Saito et al., 2019). Notably, several EDCs are considered to have adverse effects on a child’s brain, and the incidence of neurodevelopmental disorders, such as attention-deficit/hyperactivity disorder, learning disabilities, and intellectual disability, has increased over the last few decades (United States Environmental Protection Agency, 2023). Furthermore, these brain dysfunctions may be associated with disturbances in the gut microbiota due to EDC exposure (Dash et al., 2022; Gama et al., 2022; Yang et al., 2023), and the gut microbiota may be a primary or secondary target in EDC toxicity. Environmental contaminants such as herbicides, fungicides, and insecticides can cause dysbiosis, a condition characterized by decreased microbial diversity (Zhao et al., 2016; Aitbali et al., 2018; Jin et al., 2018). The brain and gut communicate bidirectionally via the brain–gut axis (Mayer et al., 2022), thereby, adverse effects on the brain or gut could impact the other owing to this axis. Therefore, when abnormalities in behavior or neuron counts are observed, dysbiosis may occur.

Organophosphate pesticides (OPs) are broad-range insecticides and are one of the most widely used in the world, accounting for 45% of the global pesticide market (Muller et al., 2007; Mali et al., 2022). OPs, a class of chemicals with a common toxicity mechanism, exert their toxicity by inhibiting acetylcholinesterase, causing the accumulation of acetylcholine. OPs play essential roles in food production, horticulture, and vector control, but they spread into the environment, including the air, soil, and water (Ore et al., 2023), and can elicit the aforementioned toxic effects in non-target organisms such as wildlife and humans. Epidemiological and animal studies have shown that early-life exposure to OPs can lead to behavioral impairment in mental health, attention span, learning ability, and memory (Hernandez et al., 2016; Sapbamrer and Hongsibsong, 2019).

Acephate (O,S-dimethyl-acetyl-phosphoramido-thioate) is currently one of 18 OPs in the registration review process of the Environmental Protection Agency for potential health risks, which is performed every 15 years. Exposure to acephate in drinking water poses health risks, as stated by the Environmental Protection Agency in a revised human health risk assessment released in 2023 (Godshall et al., 2023). To date, studies on acephate exposure have mainly focused on reproductive toxicity and endocrine disruption (Farag et al., 2000; Sampaio et al., 2020; Sasaki et al., 2023). To the best of our knowledge, studies on the neurotoxicity of acephate have been limited to basic reports, including those on muscle weakness, reduced brain substance levels in rodents (Spassova et al., 2000; WHO, 2005), and disruption of migration patterns in birds (Geen et al., 1984). In contrast, little research has been conducted on the effects of perinatal exposure to acephate on behavior and the gut microbiota. In addition, although sex differences exist in neurobehavioral abnormalities due to exposure to environmental chemicals (Soeiro et al., 2007; Jardim et al., 2020; Ommati et al., 2024), males predominate in behavioral experiments, with few studies examining both sexes. Since only one sex may be sensitive to certain chemical exposures, effects on both sexes should be investigated. Therefore, in this study, the effects of early-life exposure to acephate on the behavior and gut microbiota of adult male and female mice were evaluated. We examined whether the gut microbiota could be used as an indicator to assess nervous system-related toxicity in mice.

## 2 Materials and methods

### 2.1 Animals and treatments

**Figure 1:**
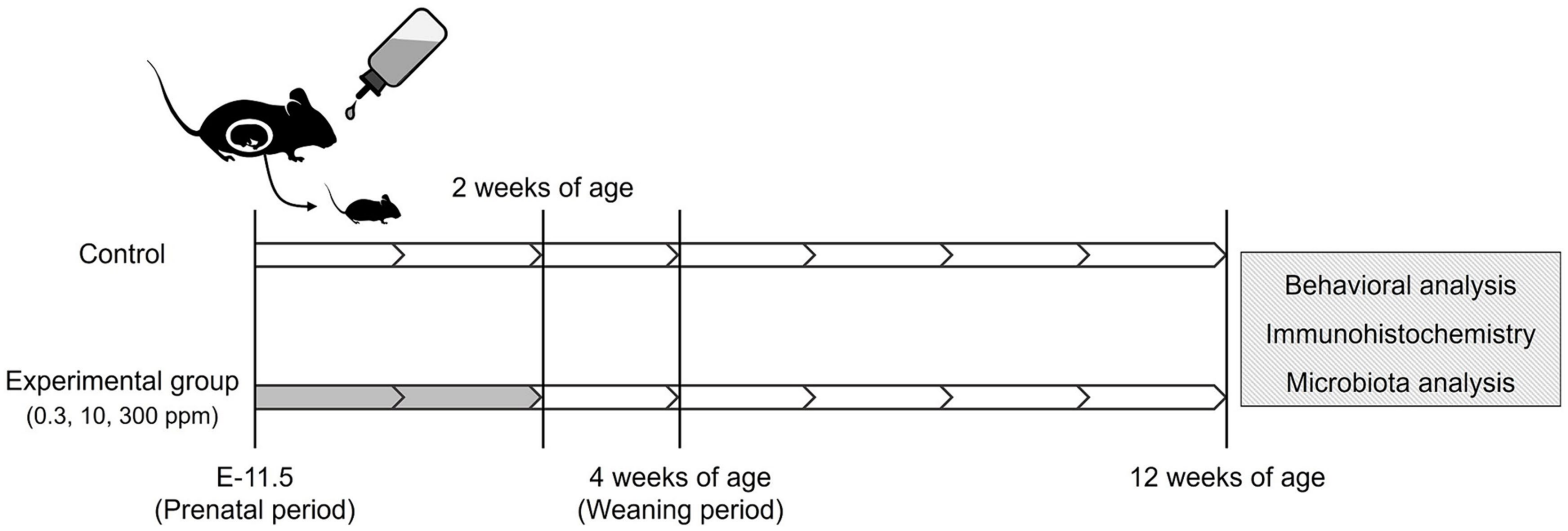
Schematic illustration of the acephate exposure protocol. White, no treatment; Gray, administration of acephate via drinking water.

Figure 1 shows the schematic workflow of the experiment. Acephate (Toronto Research Chemicals Inc., Toronto, ON, Canada) was dissolved in drinking water. Pregnant female C57BL/6 N mice at E-11 were purchased from Japan SLC (Shizuoka, Japan). We used our previous study as a reference for the concentration and exposure methods (Sasaki et al., 2023). Briefly, dams were given drinking water containing 0 (Control), 0.3 (Low-dose group), 10 (Medium-dose group), or 300 (High-dose group) ppm of acephate. Acephate-containing water was freshly prepared every 3 days. The concentration in the low-dose group was equivalent to the acceptable daily intake (ADI) registered at the Joint Food and Agriculture Organization/World Health Organization Meeting on Pesticide Residues (0.03 mg/kg/day).

The dams were exposed to acephate from the gestation period (E-11.5) to lactation when the pups were 2 weeks old. The offspring were weaned at 4 weeks of age and housed in groups of four subjects per cage by sex. To minimize animal stress, noise levels were carefully controlled in the animal rooms and adjacent areas. All animals were handled daily for 1 min for 1 week before beginning the mouse behavioral test battery.

The mice were housed in a room at a constant temperature (24 ± 1°C), humidity (60 ± 10%), and a 12-h light/dark cycle. They had free access to food (MF; Oriental Yeast Co., Ltd., Tokyo, Japan) and water. All animal care and experiments were conducted in accordance with the Regulations for Animal Experiments and Related Activities of Tohoku University. This study was approved by the Tohoku University Institutional Animal Care and Use Committee (2019noudou-004-01).

### 2.2 Behavioral test

Ten mice offspring were selected from each group with the smallest variance in body weight at 12–13 weeks of age (these mice were randomly selected from at least four litters per group). We performed behavioral tests consisting of open field test (OF), light/dark transition test (LD), and contextual/cued fear conditioning test (FZ). These experiments were conducted based on previous reports (Saito et al., 2019; Sasaki et al., 2021), with minor modifications (the strength and frequency of electric shock were changed). The measured values and images were obtained using the Image OF2, Image LD2, and Image FZ2 software (O’Hara & Co., Ltd., Tokyo, Japan) developed using the public domain ImageJ program. All experiments were conducted between 11:00 and 15:00 h. The experiments were performed in a soundproof box (78 × 63 × 65 [H] cm) made of white wood equipped with an audio speaker and a light source. Background noise during the experiments was approximately 50 dB. After each test, the apparatus was cleaned with water and wiped dry.

#### 2.2.1 Open field test

Locomotor activity was measured for 10 min using an OF apparatus consisting of white plastic material [50 × 50 × 30 (H) cm]. The LED light system was positioned 50 cm above the center of the field (25 lx at the center of the field). The total distance traveled, time spent in the center region, moving speed, and number of moving episodes were recorded using a charge-coupled device (CCD) camera positioned above the center of the OF apparatus.

#### 2.2.2 Light/dark transition test

The apparatus consisted of a cage (21 × 42 × 25 [H] cm) divided into two chambers by a partition with an opening. One chamber was made of white plastic and brightly illuminated (250 lx, light box). The other chamber, which was made of black plastic, was dark (5 lx, dark box). The mice were placed in the dark box and allowed to move freely between the two chambers through the opening for 5 min. The total distance traveled in the light box, time spent in the light box, number of transitions, and latency to enter the light box were measured using a CCD camera positioned above each chamber.

#### 2.2.3 Contextual/cued fear conditioning test

The apparatus for this experiment consisted of a conditioning chamber (test chamber: 17 × 10 × 10 [H] cm) made of clear plastic with a ceiling. The chamber floor had stainless steel rods (2 mm in diameter) spaced 5 mm apart, which delivered an electric shock to the feet of the mice. The inner wall of the chamber was covered with black and white stripes. The LED light system was positioned approximately 50 cm above the chamber (50 lx at the center of the floor). The behavior was measured using a CCD camera positioned above the chamber center. During the conditioning trial, mice were individually placed in the conditioning chamber and, after 90 s, were given three tone–shock pairings (30 s of tone at 65 dB, followed by 3 s of 1.11 mA electric shock), each separated by 120 s. Thereafter, the mice were returned to their home cages. Two days later, as a contextual fear test, the mice were returned to the conditioning chamber for 6 min without tone or shock. Two days later, for the cued fear test, they were placed in a novel chamber (with a different design and lacking black and white plastic stripes and stainless steel rods). After 3 min, a conditioning tone (with no shock) was applied for 3 min. The freezing response of the mice was measured using Image FZ2 as a consecutive 2-s period of immobility. The freezing rate (%) was calculated as follows: (freezing/session time) × 100.

### 2.3 Sample collection

At 13 weeks of age, the mice were euthanized after anesthesia. We used an anesthetic mixture of medetomidine (0.3 mg/kg, Medetomin injection Meiji; Meiji Seika Pharma Co., Ltd., Tokyo, Japan), midazolam (4.0 mg/kg, Midazolam Injection Sandoz; Sandoz K. K., Tokyo, Japan), and butorphanol tartrate (5.0 mg/kg, Vetorphale; Meiji Seika Pharma Co., Ltd., Tokyo, Japan). We used a previous study as a reference for the perfusion method (Sasaki et al., 2023). Briefly, under deep anesthesia, rapid thoracotomy was performed, followed by left intracardiac perfusion with saline and simultaneous exsanguination from the right atrium. The brains were surgically removed, fixed with methacarn solution (methanol: chloroform: acetic acid = 6:3:1), treated with ethanol and xylene, and embedded in paraffin, then sectioned into 10-μm thick sagittal sections. Fecal samples were collected from the rectum of the mice and stored in sterile tubes at −80°C until analysis.

### 2.4 Immunohistochemical analysis

Tissue sections (10-μm thick) were deparaffinized with xylene, rehydrated with ethanol (100, 95, 90, 80, and 70%), rinsed with distilled water, and incubated with HistoVT One (Nacalai Tesque, Kyoto, Japan) at 90°C for 30 min. Thereafter, they were kept at 4°C for 1 h in Blocking One (Nacalai Teque), followed by an overnight incubation at 4°C with primary antibodies. The following primary antibodies were used: rabbit polyclonal anti-doublecortin (DCX; Abcam, Cambridge, United Kingdom; ab18723; diluted 1:200), rabbit polyclonal anti-neuronal nuclei (NeuN; Novus Biologicals, Littleton, CO, United States; NBP1-77686; diluted 1:300), goat polyclonal anti-SRY-related HMG-box gene 2 (Sox2; Novus Biologicals; AF2018; diluted 1:200), and goat polyclonal anti-glial fibrillary acidic protein (GFAP; Novus Biologicals; NB100-53809; diluted 1:200). After rinsing with phosphate-buffered saline, immunoreactive elements were visualized following treatment with Alexa Flour 488-labeled anti-rabbit, and Alexa Fluor 555-labeled anti-rabbit or anti-goat secondary antibodies (Invitrogen, Waltham, MA, United States; diluted 1:1000) for 2 h at 4°C. The nuclei were stained with Hoechst 33342 (Nacalai Tesque; diluted 1:5000). For all dilutions, antibodies or Hoechst 33342 were added to the Blocking One and phosphate-buffered saline mixture. Images were obtained using an FV3000 confocal laser scanning microscope (Olympus) and analyzed using the CellSens software (Olympus).

### 2.5 Cell counting

**Figure 2:**
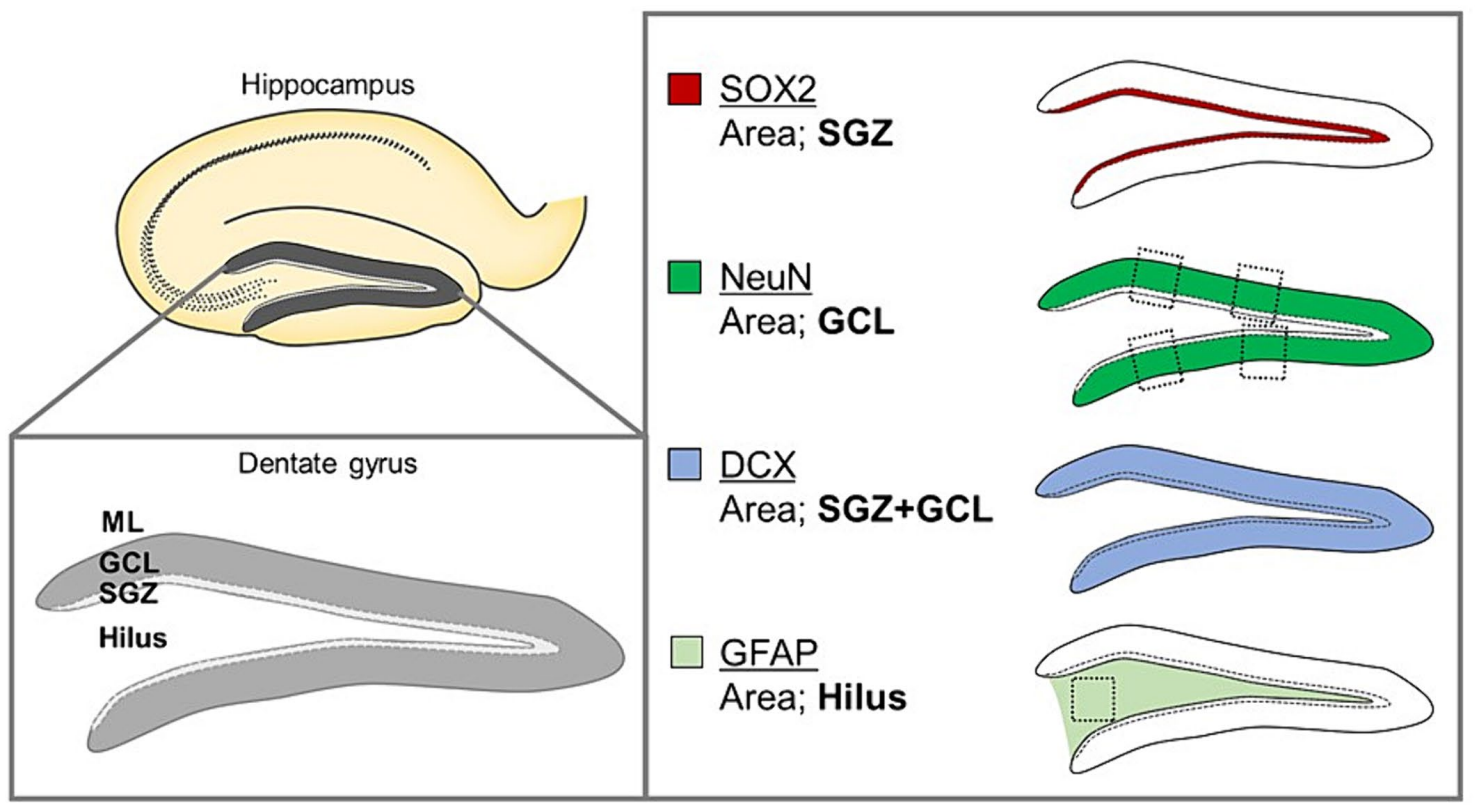
Schematic organization of the anatomical structure of the DG, including the ML, GCL, and SGZ, and the hilus and sagittal brain sections analyzed using image quantification.

To quantify each cell type, we examined three noncontiguous sagittal sections per mouse at the same anatomical level. The quantified sites and specific markers of these cells in the sagittal brain sections used in this analysis were determined with reference to previous studies (Oishi et al., 2016; Saito et al., 2019; Figure 2). For the stained images, Sox2-positive (a neural stem cell marker), DCX-positive (an immature neuron marker), NeuN-positive (a mature neuron marker), and GFAP-positive (an astrocyte marker) cells were counted under a confocal microscope using a 100- × objective. The entire subgranular zone (SGZ) of the DG was examined to quantify Sox2-positive cells. The number of DCX-positive cells was counted in the entire SGZ and granule cell layer (GCL) of the DG. The number of NeuN-positive cells was calculated from the average of four areas (two areas each on the upper and lower sides of the DG), each 50 μm wide. The number of GFAP-positive cells in the hilar region (100 × 100 μm/field) was determined.

### 2.6 Metagenomics sequencing

Fecal bacterial genomic DNA was extracted using Norgen Stool DNA Isolation Kits according to the manufacturer’s instructions. Specifically, gene amplification of the V3-V4 region of the bacterial 16S ribosomal RNA (rRNA) and 16S rRNA library preparation were performed as previously described (Suyama and Matsuki, 2015; Usami et al., 2021; Islam et al., 2022). Next-generation sequencing was performed on a MiSeq platform (Illumina) using the MiSeq reagent kit v2 (500 cycles) (Usami et al., 2021). Microbiome sequencing data were analyzed using the software package Quantitative Insights into Microbial Ecology 2 (QIIME 2, version 2021.2) according to the suggested workflow (Bolyen et al., 2019). Briefly, demultiplexed sequences were processed using the DADA2 pipeline (q2-dada2 plugin), and unique amplicon sequence variants (ASVs) were assigned a taxonomy against Greengenes 13_8 99% OTUs^1^ (McDonald et al., 2012). The 16S rRNA read counts obtained from the QIIME2 platform were further analyzed using Microbiomeanalyst 2.0 to get the abundance profiling (Lu et al., 2023).

### 2.7 Statistical analysis

Data are presented as mean ± standard error (S.E). Statistical significance was set at *p* < 0.05 (*p < 0.05). The results were compared using a one-way (factor; dose, level; each group) analysis of variance (ANOVA), followed by Dunnett’s multiple comparison tests. α- and β-diversity metrics and statistical values were generated using the QIIME2 diversity core-metrics-phylogenetic method. Microbial differential abundance was calculated using the linear discriminant analysis effect size (LEfSe) method with default settings (Segata et al., 2011). Other statistical analyses were performed using KyPlot 6.0 version 6.0.2 (KyensLab Inc., Tokyo, Japan).

## 3 Results

### 3.1 Clinical observations and body weights of offspring

During the experimental period, no clinical signs (e.g., stained eyes and nostrils or trembling) were observed in the offspring or dams. In the high-dose group, hydrocephalus was observed in one female mouse at necropsy; therefore, this mouse was excluded from the analysis. Body weights were measured weekly from 3 to 13 weeks of age. No significant differences were noted in body weights between the experimental groups and their respective control groups (data not shown).

### 3.2 Behavioral analysis

**Figure 3:**
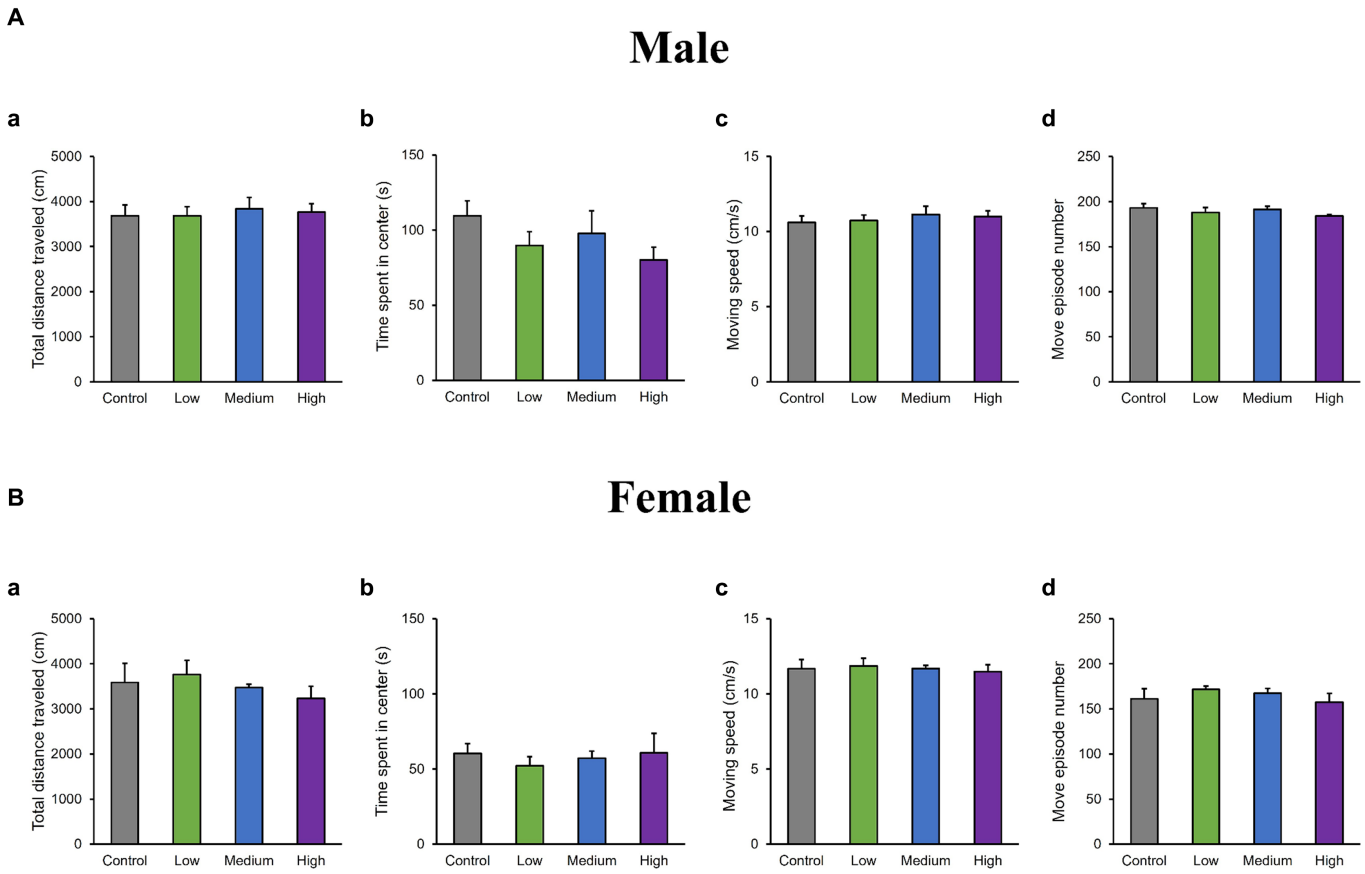
Results of the open field (OF) test. Representative scores of the OF test (total test time: 600 s) are shown. Aa and Ba: distance traveled (cm). Ab and Bb: time spent in the central area (s). Ac and Bc: moving speed (cm/s), where Ad and Bd represent the number of movements. Control: control group (male, *n* = 10; female, *n* = 10), Low: low-dose group (male, *n* = 10; female, *n* = 10), Medium: medium-dose group (male, *n* = 10; female, *n* = 10), High: high-dose group (male, *n* = 10; female, *n* = 9).

**Figure 4:**
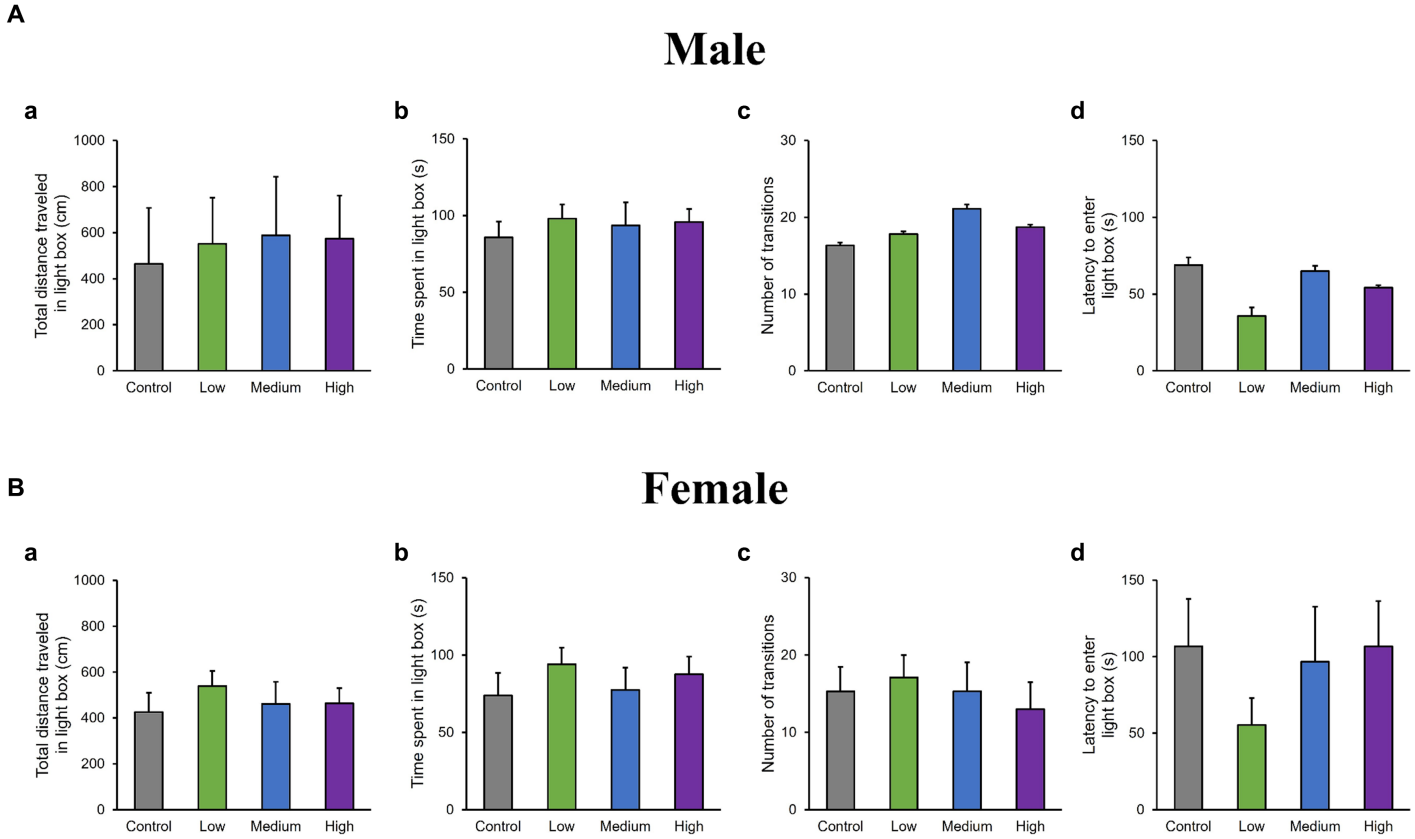
Results of the light/dark transition (LD) test. Representative scores of the LD test (total test time, 300 s) are presented. Aa and Ba: distance traveled in the light area (cm). Ab and Bb: time spent in the light area (s). Ac and Bc: number of transitions between the dark and light areas (s). Ad and Bd: latency to enter the light area (s). Control: control group (male, *n* = 10; female, *n* = 10), Low: low-dose group (male, *n* = 10; female, *n* = 10), Medium: medium-dose group (male, *n* = 10; female, *n* = 10), High: high-dose group (male, *n* = 10; female, *n* = 9).

**Figure 5:**
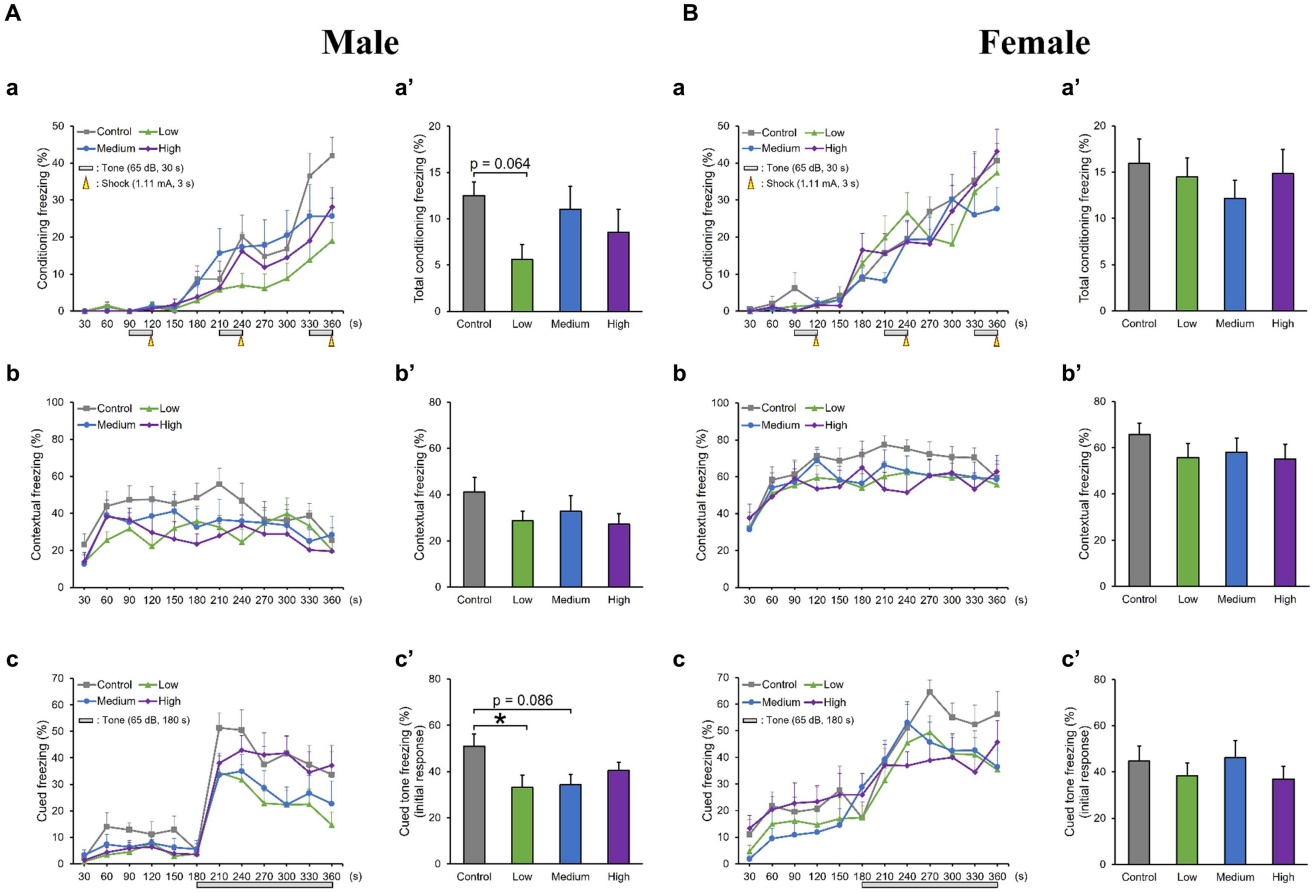
Results of the contextual/cued fear conditioning (FZ) test. (Aa, a′, Ba, a′) A conditioning test is conducted to analyze the effects of acephate on learning and contextual memory, including place (test box) and sound (cued tone). The time course of the freezing scores (%) is plotted for the conditioning test, and the average total freezing scores (%) of the control and acephate-exposed groups in the conditioning test are displayed. (Ab, b′, Bb, b′) A contextual test is conducted to analyze the effects of acephate on place memory function. The time course of the freezing scores (%) is plotted for the contextual test, and the average total freezing scores (%) of the control and acephate-exposed groups in the contextual test are displayed. (Ac, c′, Bc, c′) The cued test is conducted to analyze the effects of acephate on cued memory function. The time course of the freezing scores (%) is plotted for the cued test, and the average freezing scores (%) of the control and acephate-exposed groups for the first 1 min following the tone are presented as an initial response to the cued tone. Control: control group (male, *n* = 10; female, *n* = 10), Low: low-dose group (male, *n* = 10; female, *n* = 10), Medium: medium-dose group (male, *n* = 10; female, *n* = 10), High: high-dose group (male, *n* = 10; female, *n* = 9).

In the OF test, there were no significant differences among groups for total distance traveled (Male [*F* (3, 36) = 0.10, *p* > 0.05]; Low, *p* = 1.00; Medium, *p* = 0.93; High, *p* = 0.99; Female [*F* (3, 36) = 0.51, *p* > 0.05]; Low, *p* = 0.95; Medium, *p* = 0.99; High, *p* = 0.76); time spent in the center (Male [*F* (3, 36) = 1.29, *p* > 0.05]; Low, *p* = 0.44; Medium, *p* = 0.79; High, *p* = 0.16; Female [*F* (3, 36) = 0.26, *p* > 0.05]; Low, *p* = 0.80; Medium, *p* = 0.98; High, *p* = 1.00); moving speed (Male [*F* (3, 36) = 0.32, *p* > 0.05]; Low, *p* = 1.00; Medium, *p* = 0.72; High, *p* = 0.85; Female [*F* (3, 36) = 0.21, *p* > 0.05]; Low, *p* = 0.99; Medium, p = 1.00; High, p = 0.99), and the number of moving episodes (Male [*F* (3, 36) = 0.95, *p* > 0.05]; Low, *p* = 0.71; Medium, *p* = 0.99; High, *p* = 0.30; Female [F (3, 36) = 0.62, *p* > 0.05]; Low, *p* = 0.67; Medium, *p* = 0.90; High, *p* = 0.98) for both male and female mice across different groups (Figures 3A,B). In the LD test, acephate exposure did not significantly affect the total distance traveled in the light box (Male [*F* (3, 36) = 0.61, *p* > 0.05]; Low, *p* = 0.71; Medium, *p* = 0.48; High, *p* = 0.56; Female [*F* (3, 36) = 0.37, *p* > 0.05]; Low, *p* = 0.61; Medium, *p* = 0.98; High, *p* = 0.97); time spent in the light box (Male [*F* (3, 36) = 0.22, *p* > 0.05]; Low, *p* = 0.79; Medium, *p* = 0.93; High, *p* = 0.88; Female [*F* (3, 36) = 0.51, *p* > 0.05]; Low, *p* = 0.56; Medium, *p* = 0.99; High, *p* = 0.81); number of transitions (Male [*F* (3, 36) = 0.51, *p* > 0.05]; Low, *p* = 0.97; Medium, *p* = 0.49; High, *p* = 0.88; Female [F (3, 36) = 0.25, *p* > 0.05]; Low, *p* = 0.96; Medium, *p* = 1.00; High, *p* = 0.93); or latency to enter the light box (Male [*F* (3, 36) = 0.29, *p* > 0.05]; Low, *p* = 0.73; Medium, *p* = 1.00; High, p = 0.96; Female [*F* (3, 36) = 0.71, *p* > 0.05]; Low, *p* = 0.46; Medium, *p* = 0.99; High, *p* = 1.00) between the exposure and control groups (Figures 4A,B). In the FZ test, the freezing rate in the conditioning test decreased in the male low-dose group, although not significantly [*F* (3, 36) = 2.12, *p* > 0.05] (Low, *p* = 0.06; Medium, *p* = 0.93; High, *p* = 0.40; Figure 5A, a,a’). There was no significant difference in the freezing rate in the contextual test for males [*F* (3, 36) = 1.24, *p* > 0.05] (Low, *p* = 0.29; Medium, *p* = 0.58; High, *p* = 0.21; Figure 5A, b,b’). In the cued test, there was a significant decrease in the freezing rate in the male low-dose group and a decrease in the male medium-dose group, although these changes were not statistically significant [*F* (3, 36) = 3.55, *p* < 0.05] (Low, *p* = 0.03; Medium, *p* = 0.09; High, *p* = 0.95; Figure 5A, c,c’). Conversely, no significant behavioral changes were observed in the females in the conditioning test [*F* (3, 36) = 0.48, *p* > 0.05] (Low, *p* = 0.94; Medium, *p* = 0.51; High, *p* = 0.97), contextual test [*F* (3, 36) = 0.69, *p* > 0.05] (Low, *p* = 0.49; Medium, *p* = 0.67; High, *p* = 0.47), or cued test [*F* (3, 36) = 0.52, *p* > 0.05] (Low, *p* = 0.81; Medium, *p* = 1.00; High, *p* = 0.72; Figure 5B).

### 3.3 Histopathological and immunohistochemical analysis

**Figure 6:**
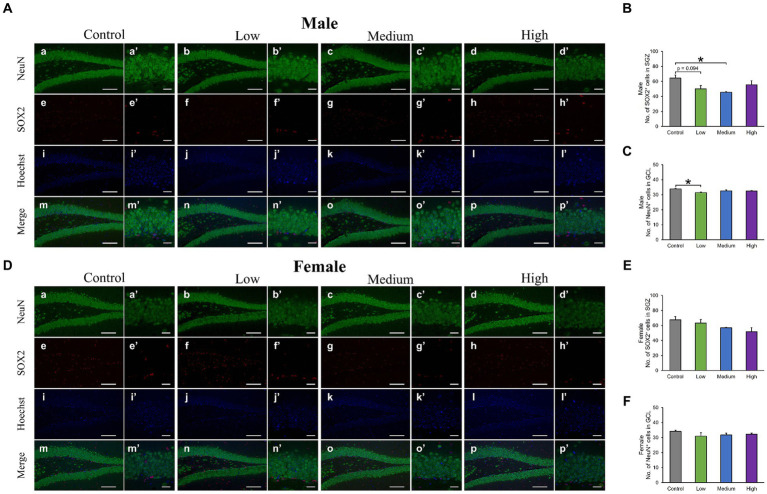
Immunohistochemical analysis with neuronal nuclei (NeuN) and SRY-related HMG-box 2 (Sox2). **(A,D)** Male or female representative images of NeuN (green), Sox2 (red), and Hoechst (blue) in the DG of 13-week-old control and acephate-exposed mice. Scale bars, 100 μm (Aa-p, Da-p), 20 μm (Aa′-p′, A a′-p′). **(B,C,E,F)** The number of NeuN- or Sox2-positive cells in the male or female of each group. Control: control group (male; Aa, a′, e, e′, i, i′, m, m′: female; Da, a′, e, e′, i, i′, m, m′), Low: low-dose group (male; Ab, b′, f, f′, j, j′, n, n′: female; Db, b′, f, f′, j, j′, n, n′), Medium: medium-dose group (male; Ac, c′, g, g′, k, k′, o, o′: female; Dc, c′, g, g′, k, k′, o, o′), High: high-dose group (male; Ad, d′, h, h′, l, l′, p, p′: female; Dd, d′, h, h′, l, l′, p, p′), *n* = 3 per group.

**Figure 7:**
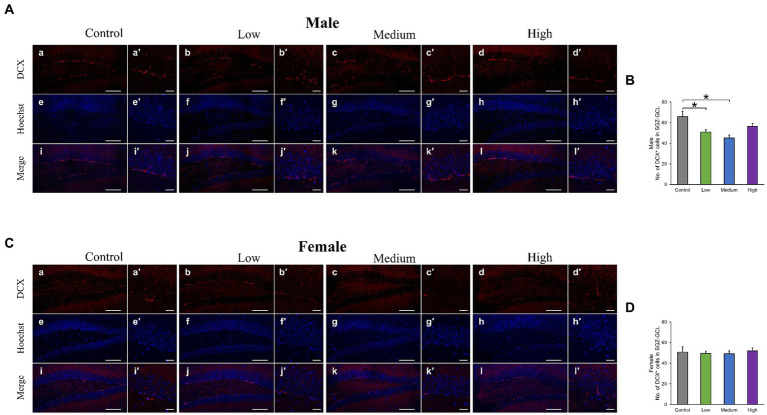
Immunohistochemical analysis with doublecortin (DCX). **(A,C)** Male or female representative images of DCX (red) and Hoechst (blue) in the DG of 13-week-old control and acephate-exposed mice. Scale bars, 100 μm (Aa-l, Ca-l), 20 μm (Aa′-l′, Ca′-l′). **(B,D)** The number of DCX-positive cells in the male or female of each group. Control: control group (male; Aa, a′, e, e′, i, i′: female; Ca, a′, e, e′, i, i′), Low: low-dose group (male; Ab, b′, f, f′, j, j′: female; Cb, b′, f, f′, j, j′), Medium: medium-dose group (male; Ac, c′, g, g′, k, k′: female; Cc, c′, g, g′, k, k′), High: high-dose group (male; Ad, d′, h, h′, l, l′: female; Cd, d′, h, h′, l, l′), *n* = 3 per group.

**Figure 8:**
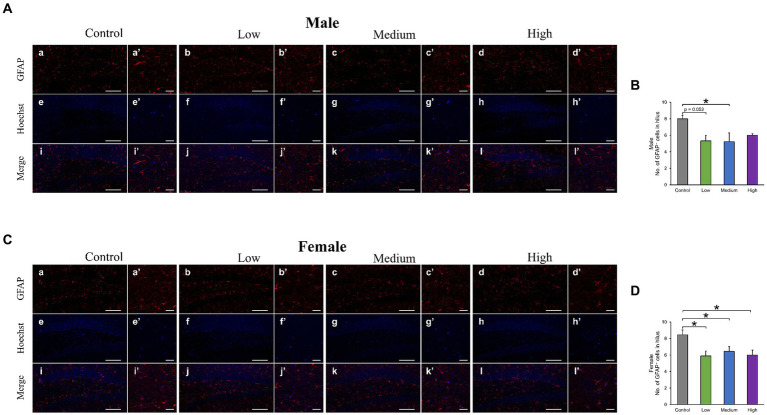
Immunohistochemical analysis with glial fibrillary acidic protein (GFAP). **(A,C)** Male or female representative images of GFAP (red) and Hoechst (blue) in the DG of 13-week-old control and acephate-exposed mice. Scale bars, 100 μm (Aa-l, Ca-l), 20 μm (Aa′-l′, Ca′-l′). **(B,D)** The number of GFAP-positive cells in the male or female of each group. Control: control group (male; Aa, a′, e, e′, i, i′: female; Ca, a′, e, e′, i, i′), Low: low-dose group (male; Ab, b′, f, f′, j, j′: female; Cb, b′, f, f′, j, j′), Medium: medium-dose group (male; Ac, c′, g, g′, k, k’: female; Cc, c′, g, g′, k, k′), High: high-dose group (male; Ad, d′, h, h′, l, l′: female; Cd, d′, h, h′, l, l′), *n* = 3 per group.

In the H&E-stained hippocampus, no histopathological abnormalities (i.e., neuronal cell death) were observed (data not shown). The male low-dose as well as medium-dose groups exhibited significantly less number of Sox2-positive cells than the control group [*F* (3, 8) = 3.84, *p* < 0.05] (Low, *p* = 0.09; Medium, *p* = 0.03; High, *p* = 0.36; Figures 6A,B). The number of NeuN-positive cells was significantly lower in the male low-dose group than in the control group [F (3, 8) = 4.27, *p* < 0.05] (Low, *p* = 0.87; Medium, *p* = 0.34; High, *p* = 0.12; Figures 6A,C). However, the counts of Sox2- [*F* (3, 8) = 2.07, *p* > 0.05] (Low, *p* = 0.02; Medium, *p* = 0.23; High, *p* = 0.16) and NeuN-positive cells [*F* (3, 8) = 1.03, *p* > 0.05] (Low, *p* = 0.28; Medium, *p* = 0.48; High, *p* = 0.65) in females did not significantly differ across all treated groups compared to those in the controls (Figures 6D–F). Additionally, the quantification of DCX-positive cells showed significantly fewer immature neurons in the male low- and medium-dose groups than in the control group [*F* (3, 8) = 6.56, *p* < 0.05] (Low, *p* = 0.04; Medium, *p* = 0.01; High, *p* = 0.19) (Figures 7A,B). Similar to the other results, the number of DCX-positive cells in females was not significantly different among the groups [*F* (3, 8) = 0.19, *p* > 0.05] (Low, *p* = 0.98; Medium, *p* = 0.96; High, *p* = 0.98; Figures 7C,D). Moreover, in the male low-dose group, the number of GFAP-positive cells was lower; the male medium-dose group displayed a significant decrease in the number of GFAP-positive cells compared to the control group [*F* (3, 8) = 0.06, *p* < 0.05] (Low, *p* = 0.05; Medium, *p* = 0.04; High, *p* = 0.15; Figures 8A,B). Notably, we observed a significant reduction in the number of GFAP-positive cells in females in all treated groups compared to that in the control group [*F* (3, 8) = 0.01, *p* < 0.05] (Low, *p* = 0.01; Medium, *p* = 0.04; High, *p* = 0.01; Figures 8C,D). No morphological differences were observed in GFAP-positive cells.

### 3.4 Microbiota analysis

**Figure 9:**
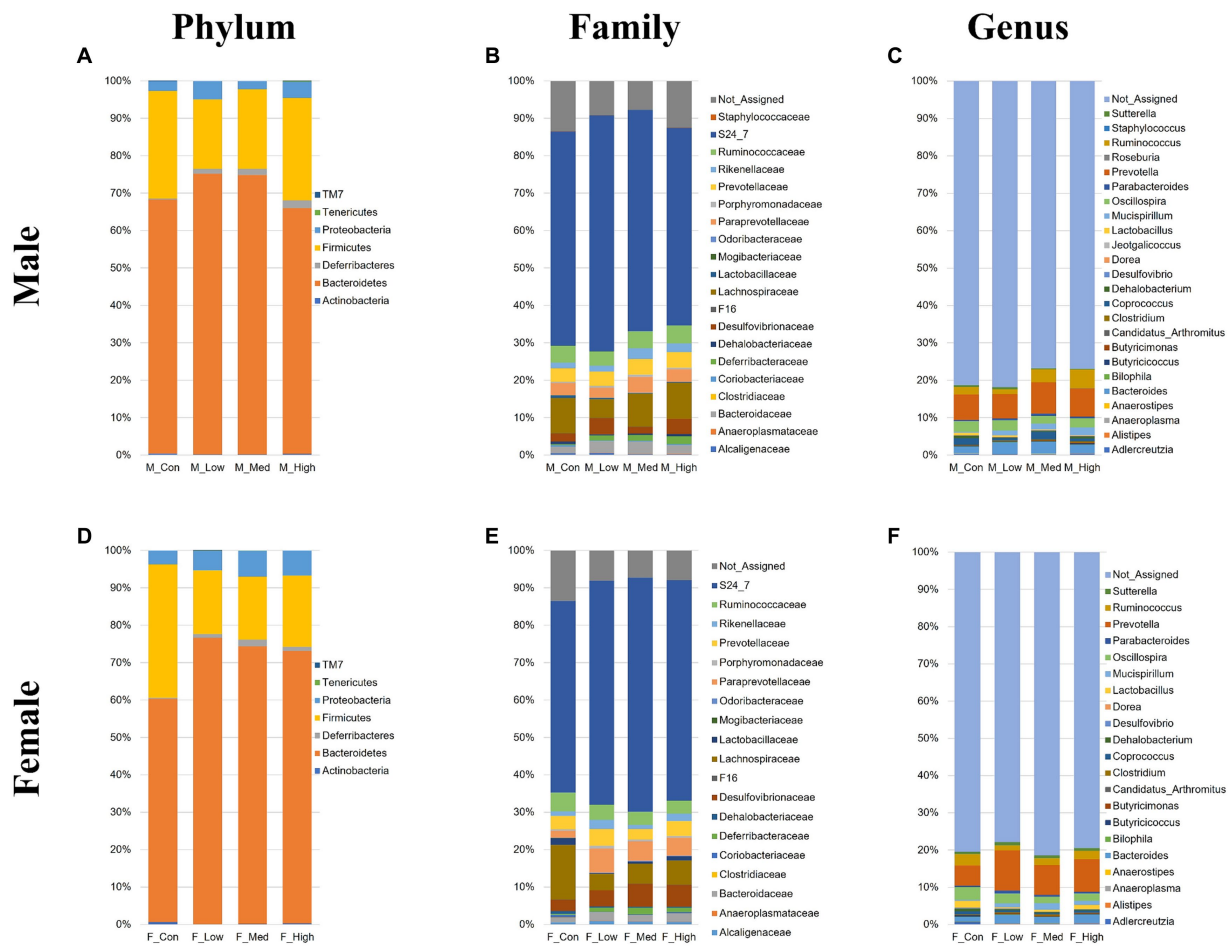
Microbial composition at the phylum, family, and genus levels. Male or female microbial compositions at the phylum **(A,D)**, family **(B,E)**, and genus levels **(C,F)**. M_Con, male control group; M_Low, male low-dose group; M_Med, male medium-dose group; M_High, male high-dose group; F_Con, female control group; F_Low, female low-dose group; F_Med, female medium-dose group; F_High, female high-dose group (*n* = 3 per group).

**Figure 10:**
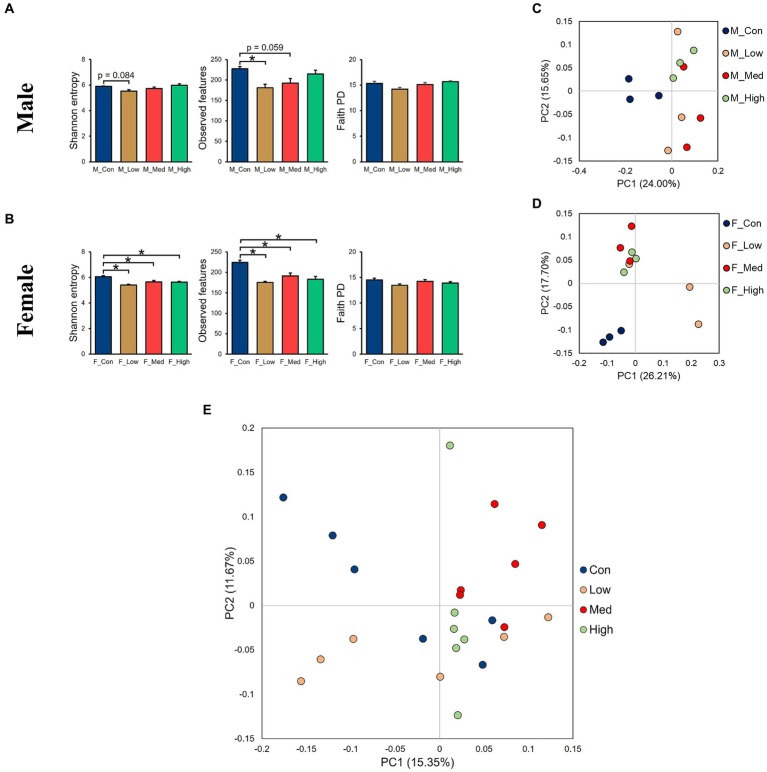
Alpha- and beta-diversity comparisons of the gut microbiomes of each group. **(A,B)** Alpha-diversity index based on Shannon’s index, observed features, and faith phylogenetic diversity categorized by sex. **(C,D)** Principal Coordinate Analysis based on the unweighted distance matrix of the bacterial 16S rRNA amplicon sequencing data for fecal samples categorized by sex, **(E)**, and by dose. M_Con: male control group, M_Low: male low-dose group, M_Med: male medium-dose group, M_High: male high-dose group, F_Con: female control group, F_Low: female low-dose group, F_Med: female medium-dose group, F_High: female high-dose group (*n* = 3 per group). Con: control group, Low: low-dose group, Med: medium-dose group, High: high-dose group (*n* = 6 per group).

**Figure 11:**
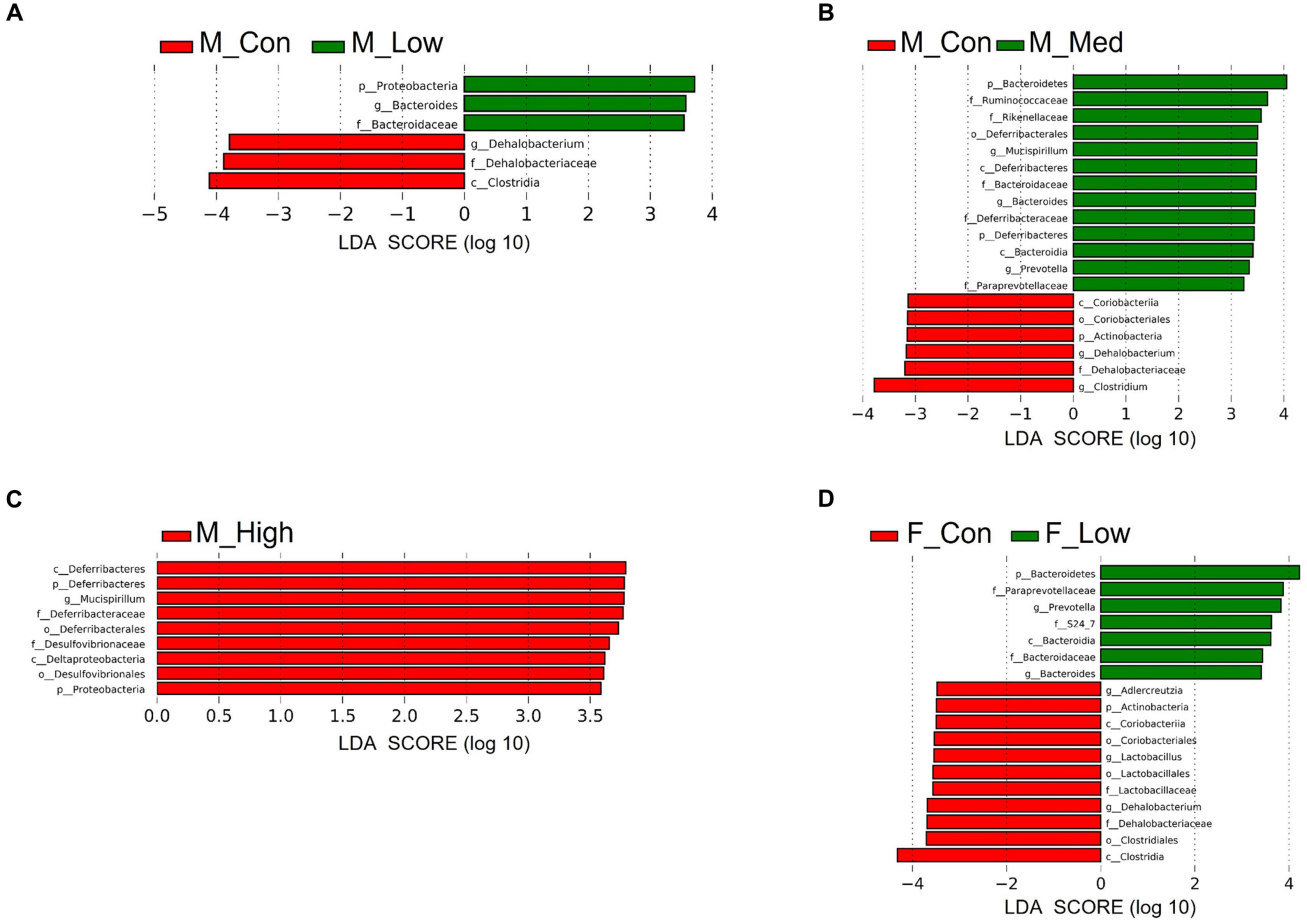
Identification of differential abundant microbiota. Linear discriminant analysis (LDA) scores of differential abundant taxa phylum (p), class (c), order (o), family (f), and genera (g) in each group. Low: low-dose group (male; A: female; D), Medium: medium-dose group (male; B: female; E), High: high-dose group (male; C: female; F). M_Con, male control; M_Low, male low-dose; M_Med, male medium-dose; M_High, male high-dose; F_Con, female control; F_Low, female low-dose (*n* = 3 per group).

Regardless of the fecal sample, Bacteroidetes, Firmicutes, and Proteobacteria dominated the microbial taxa at the phylum level (Figures 9A,D). At the family and genus levels, the abundance of *S24-7* and *Prevotella* was consistently high across all groups (Figures 9B,C,E,F). In addition, approximately 10% of the microbial taxa were not assigned at the family level, and more than 70% of the microbial taxa were not assigned at the genus level. Based on α-diversity analysis, Shannon’s index (richness and evenness) [Male *F* (3, 8) = 3.99, *p* > 0.05]; Low, *p* = 0.08; Medium, *p* = 0.52; High, *p* = 0.83, Female [*F* (3, 8) = 10.95, *p* < 0.01]; Low, *p* = 0.001; Medium, *p* = 0.02; High, *p* = 0.01, observed features (no. of identified OTUs) [Male *F* (3, 8) = 5.59, *p* < 0.05]; Low, *p* = 0.02; Medium, *p* = 0.06; High, *p* = 0.64, Female [*F* (3, 8) = 13.77, *p* < 0.01]; Low, *p* = 0.0009; Medium, *p* = 0.01; High, *p* = 0.003, and faith (phylogenetic diversity) [Male *F* (3, 8) = 3.35, *p* > 0.05]; Low, *p* = 0.12; Medium, *p* = 0.94; High, *p* = 0.80, Female [*F* (3, 8) = 2.16, *p* > 0.05]; Low, *p* = 0.10; Medium, *p* = 0.89; High, *p* = 0.44 indices showed a decrease in diversity in each experimental group compared to that in their respective control groups in both sexes (Figures 10A,B). Principal Coordinate Analysis of β-diversity was conducted based on unweighted UniFrac distances. The Principal Coordinate Analysis plot revealed an overall statistical difference (*p* < 0.001), although no statistical difference was observed in the pairwise PERMANOVA (Figures 10C,D). However, clear clustering differences by dose, including both sexes, were evident (Figure 10E), and pairwise PERMANOVA yielded significant differences between comparisons, except between the medium and high doses (Table 1). Differences in the abundance of microbiota between the control and experimental groups were investigated using the LEfSe. The relative abundance of Proteobacteria was higher in the male low-dose group (Figure 11A), that of Bacteroidetes and Deferribacteres was highest in the medium-dose group (Figure 11B), and that of Deferribacteres and Proteobacteria was highest in the high-dose group (Figure 11C) compared to that in the control group. The relative abundance of Bacteroidetes was higher in the female low-dose group, whereas Actinobacteria were more abundant in the control group (Figure 11D). However, there were no significant differences in bacterial abundance between the female medium- and high-dose groups.

**Table 1:** Results of pairwise PERMANOVA tests.

Comparison groups	Pseudo-F	*p*-value	Q-value
M_Con vs. M_Low	1.842868	0.112	0.224
M_Con vs. M_Med	2.560965	0.102	0.224
M_Con vs. M_High	2.489776	0.106	0.224
M_Low vs. M_Med	1.155649	0.307	0.307
M_Low vs. M_High	1.318092	0.209	0.307
M_Med vs. M_High	1.222952	0.298	0.307
F_Con vs. F_Low	2.593436	0.105	0.126
F_Con vs. F_Med	2.635604	0.103	0.126
F_Con vs. F_High	2.186985	0.102	0.126
F_Low vs. F_Med	2.060111	0.094	0.126
F_Low vs. F_High	1.833455	0.092	0.126
F_Med vs. F_High	1.467265	0.218	0.218
Con vs. Low	2.847436466	0.004	0.008
Con vs. Med	3.102858663	0.002	0.008
Con vs. High	2.70044962	0.003	0.008
Low vs. Med	1.65562931	0.024	0.036
Low vs. High	1.494325773	0.037	0.0444
Med vs. High	1.244483399	0.14	0.14

M_Con, male control group, M_Low, male low-dose group; M_Med, male medium-dose group; M_High, male high-dose group; F_Con, female control group; F_Low, female low-dose group; F_Med, female medium-dose group; F_High, female high-dose group (*n* = 3 per group). Con, control group; Low, low-dose group; Med, medium-dose group; High, high-dose group (*n* = 6 per group).

## 4 Discussion

This study investigated the effects of early acephate exposure on the CNS in adult male and female mice. In addition, we evaluated whether the gut microbiota could be used as an indicator to determine the neurotoxicity.

In the OF test, there were no significant changes in the total distance traveled, moving speed, and number of moving episodes for both sexes. These results suggest that acephate exposure does not produce dramatic changes in basic locomotor activity. Additionally, more anxious mice typically spend less exploratory time in the center of the apparatus in the OF test (Carter and Shieh, 2015) and in brightly lit spaces in the LD test (Takao and Miyakawa, 2006). In this study, the time spent in the central region of the OF test and all evaluations in the LD test showed no changes. Therefore, the results suggest that acephate exposure does not induce anxiety related to novel environments or brightly lit spaces. Exposure to insecticides and herbicides can cause motor deficits, and rodents exposed to insecticides and herbicides are often used as models for Parkinson’s disease (Schober, 2004). However, the phenotype of motor activity is not always consistent across studies. In previous studies with OPs, repeated exposure to methyl parathion impaired motor functions in adult rats (Sun et al., 2006), a single dose of chlorpyrifos during the developmental period increased total traveled distance in the open field test (Ricceri et al., 2006), and perinatal exposure to nicotine and diazinon had no effect on the motor activity in the open field test (Lee et al., 2021). It is thought that the phenotype of motor activity differs depending on the route of administration (i.e., oral administration, intraperitoneal administration, and administration in drinking water) and the administration regime (i.e., single administration, and chronic administration). In addition, the reason for there being no change in overall motor activity with increased doses of acephate may be because acephate has the lowest level of neurotoxicity compared to that of other typical OPs (Ireland et al., 2022). Therefore, more detailed evaluations focusing on gait and movements at regular intervals are needed in the future.

In the FZ test, the male low- and medium-dose groups showed a significant decrease in their freezing rate or a tendency to decrease it. In the male low-dose group, conditioning and cued freezing rates were low. This result suggests that cued learning, short-term memory formation, and cued memory retrieval were impaired in the male low-dose group. Additionally, the male medium-dose group tended to have impaired cued memory retrieval. Thus, rather than the different degrees of abnormalities appearing at different doses as behavioral effects of early-life acephate exposure, qualitatively different abnormalities are thought to appear. In a previous study examining the behavioral effects of phenylalkylamine and indoleamine psychedelics, mouse behavior was not dose-dependent (Chen et al., 2023), which is consistent with the results of the present study. The FZ test was selected for this study due to its high throughput and significant dependence on the hippocampus (Phillips and LeDoux, 1992). However, this test involves associative learning, which is influenced by anxiety and/or fear resulting from psychological stress. To assess effects on pure memory, it is advisable to include additional analyses such as the Y-maze and radial arm maze.

Neural stem cells can self-renew and differentiate into several types of neurons and glial cells. The perinatal stage (critical period) is a period of active neurogenesis and is very important in terms of neural circuit organization and functional maturation of the brain; thus, environmental factors (e.g., chemical exposure and stress) during development may interfere with normal brain function development. In addition, although the frequency of neurogenesis declines after puberty, it is continuous throughout life in the SGZ of the hippocampal DG region, and dysregulation of neurogenesis after maturation is associated with cognitive decline and psychiatric symptoms (Toda et al., 2019). Reduced neurogenesis in adult mice is associated with increased anxiety-like behavior and impaired fear memory recall (Imayoshi et al., 2008; Revest et al., 2009). In experiments using behavioral tests and cell count methods similar to those used in this study, early-life exposure to neonicotinoid insecticides resulted in memory impairment and a decrease in SOX2-positive cells in the DG (Saito et al., 2023). In this study, an overall decrease in the number of neural stem cells, immature neurons, and mature neurons was observed in the male treatment group, indicating reduced neurogenesis. In other words, early-life exposure to acephate may have triggered a reduction in the number of neurons at each stage of differentiation, starting with a decrease in the pooled number of neural stem cells.

In addition, astrocytes play diverse roles as housekeeping cells, such as forming the blood–brain barrier, maintaining homeostasis of the extracellular environment, taking up neurotransmitters, and supplying energy to neurons (Verkhratsky et al., 2021). In particular, hippocampal astrocytes are thought to be responsible for the regulation of anxious behavior, learning, and memory (Akther and Hirase, 2022; Cho et al., 2022), and a decrease in astrocyte number has been found to induce affective disorders (Czéh et al., 2006). In this study, both neuron and astrocyte numbers were reduced in all-male treatment groups, suggesting that the behavioral phenotype of male mice reflects the inadequate construction of neural circuits involved in learning, memory, and emotion. In contrast, no behavioral abnormalities were observed in females despite the reduced number of astrocytes in all treatment groups. This result may be due to the detection limit of the three behavioral analyses conducted in this study and the possibility that it would become apparent as a behavioral abnormality over time. Furthermore, Wnt, BMP, Notch, and STAT signaling are essential for neuronal cell fate decisions, such as neurogenesis and astrogenesis (Wen et al., 2009). Therefore, in the male and female treatment groups, there is likely an imbalance in the signals that induce differentiation into neurons and astrocytes, regardless of whether the final output, that is, behavior, is abnormal.

Although the reason for these behavioral changes remains unclear, it is possible that changes in the gut microbiota also mediate adverse effects on the nervous system. Accumulating evidence supports the possibility that gut microbiota may influence the initiation and regulation of neurogenesis (Cerdó et al., 2020). The gut microbiota established at birth increases in diversity during development and transitions into a stable state during maturation under the influence of sex hormones (Kim et al., 2020). Disturbances in the developing gut microbiota also have a significant impact on neurodevelopment. In a juvenile mouse model of enterotoxemia treated with antibiotics, neurogenesis was disturbed in the hippocampus, and behavioral deficits were induced (Liu et al., 2022). The mechanisms by which the gut microbiota modulates neurogenesis in the adult hippocampus have been shown to include pathways that alter hippocampal brain-derived neurotrophic factor (BDNF) levels via the vagus nerve, the immune system via cytokines and microglia, short-chain fatty acids, a metabolite of gut bacteria, and the endocrine system through the regulation of the HPA axis (Guzzetta et al., 2022). The gut microbiota analysis in this study showed a decrease in α-diversity in both males and females and different β-diversity in each group. LEfSe analysis to confirm the abundance of bacterial species showed a significant variation among the males in all treatment groups, whereas in females, a significant variation was only observed in the female low-dose group. Additionally, LEfSe analysis showed that the characteristic bacterial species found in acephate-exposed mice were Proteobacteria, Bacteroidetes, and Deferribacteres. Exposure to polystyrene nano-plastics induces anxiety-like behavior in rats by upregulating the abundance of these species (Jiang et al., 2023). High-fat diet feeding increases the Proteobacteria population and leads to memory impairment and anxiety-like behaviors in mice (Jeong et al., 2019). Proteobacteria and Bacteroidetes were abundant in patients with Alzheimer’s who exhibited cognitive impairment (Vogt et al., 2017; Liu et al., 2019). Therefore, it is possible that the microbiota balance is disturbed, leading to an increased abundance of bacteria within these phyla and resulting in memory impairment. Overall, results for males indicated that behavior, neurogenesis, and gut microbiota were all negatively affected. In contrast, results for females showed no behavioral effects but did reveal negative effects on astrocyte counts and gut microbiota. The behavioral, immunohistological, and microbiota data obtained in this study were scattered, and sample size was insufficient to statistically prove these relationships. Therefore, it remains unclear whether the gut microbiota can be used as a biomarker for neurological deficits due to exposure to environmental chemicals. Moreover, there is a lack of toxicological data on the relationship between brain function and the gut environment, including behavior, in both humans and mice; therefore, additional evidence needs to be accumulated to elucidate the causal relationship between gut-brain-behavior.

Males were also more susceptible to acephate-induced harmful effects than females. This sex difference in vulnerability to neuropathy is consistent with a higher incidence of many neurodevelopmental disorders in men (Boyle et al., 2011). The primary reason for male vulnerability to neuropathy is hormonal influences. Estradiol, the primary female sex hormone, plays neuroprotective and neurorepairing roles in the developing brain. It increases neurogenesis and synaptic plasticity, mediates neurotrophic factors, protects and remyelinates myelin, and reduces oxidative stress and neuroinflammation (Azcoitia et al., 2019). Males typically have fewer estrogen receptors throughout the CNS than females (Vahter et al., 2007). Therefore, males may have less estradiol action than females and may be more sensitive to the adverse effects of prenatal exposure to neurotoxins. Secondly, the rate of neural maturation *in utero* varies according to sex, and the maturation of neural functions in male fetuses is slower than that in female fetuses. For example, in a previous study that observed heart rate responses to assess fetal neurodevelopment, females exhibited mature responses earlier than males (Buss et al., 2009). This difference in the maturation rate may indicate a more prolonged period of sensitivity to neurotoxins in the male brain. Further analysis of sex-specific reactivity to chemical exposure and its factors is required including gene expression.

This study has some limitations. First, the generalizability of the findings to humans may be limited because our findings were derived from mice. The short-term nature of behavioral analyses might not have fully captured the long-term effects, highlighting the need for further exploration. Notably, the observed non-dose-dependent effects add complexity to the interpretation. In addition, the difficulty in this kind of study is the physiological and behavioral changes in the maternal animals caused by chemical exposure. Although we confirmed that there were no changes in the nest building and nursing behavior of animals in this study, we cannot rule out the possibility that the offspring were affected by changes in milk composition or other maternal behavior. Therefore, it is necessary to assess whether acephate disrupts the early development of offspring, including through mother-infant interactions. Although additional research on the mechanisms by which environmental chemicals affect the CNS via the gut microbiota is required, we hope that the present study can help in understanding the brain–gut relationship with regard to toxicology.

This study demonstrated, for the first time, that acephate exposure during the developmental period interferes with neurodevelopment, particularly in male mice, induces behavioral abnormalities related to learning memory, and disrupts the gut microbiota composition. The observed sex-specific vulnerabilities in neurodevelopment owing to acephate exposure emphasize the importance of considering sex differences in understanding and addressing neurotoxic effects. Furthermore, the correlation between disrupted gut microbiota composition and neurological outcomes suggests a potential avenue for identifying interventions targeting the brain–gut axis to ameliorate the impact of environmental exposures on mental health and developmental disorders. These findings contribute to a growing body of research linking exposure to environmental chemicals to neurodevelopmental disorders, urging further exploration of preventive measures and treatment modalities with broader applicability to neurological diseases derived from chemical exposures.

## Data availability statement

The original contributions presented in the study are publicly available. This data can be found here: https://dataverse.harvard.edu/dataset.xhtml?persistentId=doi:10.7910/DVN/WY3MWM.

## Ethics statement

The animal study was approved by Tohoku University Institutional Animal Care and Use Committee. The study was conducted in accordance with the local legislation and institutional requirements.

## Author contributions

TS: Conceptualization, Data curation, Funding acquisition, Resources, Validation, Writing – original draft, Writing – review & editing. JI: Data curation, Writing – review & editing. KH: Conceptualization, Writing – review & editing. TN: Conceptualization, Writing – review & editing. KT: Conceptualization, Funding acquisition, Project administration, Resources, Writing – review & editing, Supervision.

## Footnotes

^1^ https://greengenes.lbl.gov/
